# Patterns of Neurological Adverse Events Among a Retrospective Cohort of Patients Receiving Immune Checkpoint Inhibitors

**DOI:** 10.2217/imt-2023-0273

**Published:** 2024-03-14

**Authors:** John C Hunting, Andrew T Faucheux, Sarah N Price, Catherine A Elko, Alexander Quattlebaum, Chance Bloomer, Eric Olson, William J Petty, Thomas W Lycan

**Affiliations:** 1 Department of Internal Medicine, Wake Forest School of Medicine, Winston-Salem, NC27157, USA; 2 Department of Hematology & Oncology, Wake Forest School of Medicine, Winston-Salem, NC27157, USA; 3 Department of Social Sciences & Health Policy, Wake Forest School of Medicine, Winston-Salem, NC27157, USA

**Keywords:** brain metastasis, central nervous system, immune checkpoint inhibitors, incidence, melanoma, neurological adverse events, peripheral nervous system, renal cell carcinoma

## Abstract

**Aim:**

Neurological adverse events (NAEs) are infrequent immune checkpoint inhibitor (ICI) outcomes poorly characterized in extant research, complicating their clinical management.

**Methods:**

This study characterized the frequency, severity, patterning and timing of NAEs using a large retrospective registry, including all patients who received at least one dose of an ICI from 2/1/2011–4/7/2022 within our health network.

**Results:**

Among 3137 patients, there were 54 NAEs (1.72% any grade; 0.8% grade 3–4). Most NAEs were peripheral (57.4%) versus central (42.6%). Melanoma and renal cell carcinoma were significantly associated with NAEs.

**Conclusion:**

The incidence of NAEs was rare though higher than many prior case estimates; the timing was consistent with other AEs. NAEs frequently occurred in tumor types known to favor brain metastases.

## Plain language summary

Immune checkpoint inhibitors are new drugs for cancer. They boost your body’s defenses to fight cancer cells. These drugs can be used alone or with other cancer treatments. Most people are okay with these medicines, but some might have problems in different parts of the body. This can be tricky to figure out. Rarely, there can be issues in the brain or nerves. These side effects are rare, happening in about 2 in every 100 people who use the drugs. They are more common in certain cancers like melanoma and kidney cancer. As doctors learn more about these side effects, they can better predict, treat, and prevent them.

Immune checkpoint inhibitor (ICI) therapies can improve progression-free and overall survival in patients with cancer who otherwise have poor prognoses [1,2]. In 2018, over 40% of all patients with cancer were considered eligible for immunotherapy-based treatments [3]. ICIs generally have a more favorable side effect profile than traditional therapies (e.g., chemotherapy), but they are also associated with a broad array of immune-related adverse events (AEs), that can involve any organ and range from mild to fatal. A recent meta-analysis of 125 clinical trials and the registration trials for pembrolizumab found that the majority (60–70%) of patients have at least one adverse event of any grade, though few (<10%) high grade (i.e., Common Terminology Criteria for Adverse Events [CTCAE] 3–5) [4,5]. Among these adverse events is a heterogeneous group of neurotoxicities categorized as neurological adverse events (NAE), less frequently reported than other immune-related AEs. Although the attribution of adverse events as NAE varies across studies, this broad category often includes common side effects (e.g., some types of fatigue, headaches) and rare ones (e.g., seizure, polyneuropathy, isolated nerve palsies, paresthesia, myasthenia gravis, polyradiculopathy and encephalitis) [6]. Due to their heterogeneity in presentation, timing, and severity, NAE are often challenging to diagnose and, therefore, likely under-reported [7]. In many cases, NAE are diagnoses of exclusion, which can lead to delayed diagnosis and treatment [8].

Although the incidence of NAEs will continue to increase, given the widespread use of ICIs, there has been little high-level data about them. Comparing rates of immune-related AEs across clinical trials is complicated by varied classification criteria and cut points for reporting. This problem may be especially relevant for NAE since deciding whether to include common symptoms such as headache and fatigue may strongly influence prevalence estimates. Thus, extant research on NAE has relied mainly on small case series and a few cohorts. Given these limitations, it is unsurprising that NAE incidence estimates vary widely (1–12%). As described in a review by Möhn *et al.* [7] and several other cohorts, peripheral NAE are typically more common than central NAE. A recent retrospective study using the US FDA Adverse Event Reporting System database (N = 50,406 ICI-related reports) found an incidence rate of 7.2% for neurologic AEs of any grade. In contrast, a cohort study of 500 patients estimated the incidence of all NAE as <=1% [9,10]. Higher grade NAE appear relatively infrequent. A single-center study in Korea (N = 1503) found that 0.6% of patients developed severe peripheral NAE, leading to hospitalization and even death [11]. An analysis of melanoma patients treated with nivolumab (N = 576) found an incidence rate of <1% higher grade (3–4) NAE and a rate of 12% for low-grade (1–2) NAE [12,13].

While these studies offer foundational incidence estimates, more data from cohorts treated in real-world clinical settings with consistent classification criteria are needed to understand the incidence of NAE. Additionally, most extant studies lack critical details that could inform treatment and shed light on potential mechanisms, such as risk factors and timing of events during treatment. As immunotherapy obtains new indications and the applicable population grows, so does the need to better characterize and address NAE. Our study aims to address these gaps by describing NAE from several clinical domains. Specifically, we use data from a large retrospective registry of over 3000 patients to report the overall incidence of low and high-grade NAE, the proportion of AEs with peripheral versus central nervous system involvement, correlates of NAE incidence, and the timing of events following treatment initiation.

## Methods

### Study population & design

The present study was approved by the institutional review board at Wake Forest School of Medicine. Patients were eligible for this study if they had received at least one dose of an ICI for any indication between 1 February 1 2011 and 7 April 7 2022, at either the Atrium Health Wake Forest Baptist Comprehensive Cancer Center (AHWFB-CCC) or any associated outreach clinic. Additionally, to be included in the present analyses, patients had to have complete outcome data (e.g., adverse events). A total of 3145 patients were initially included in the study, although eight patients had missing outcome data and were excluded for a final cohort of 3137. Informed consent was waived by the institutional review board. Trained clinical research specialists from Vasta Global reviewed clinical documentation from patients’ electronic health records (EHR). They entered relevant information into a secure, cloud-based REDCap registry that was developed by study investigators and validated with audits and data quality rules. Adverse event attribution and severity were estimated retrospectively using National Cancer Institute guidelines and the Common Terminology Criteria for Adverse Events [14].

### Study variables

Data collected from the EHR included age, race, gender, comorbidities (prior acute respiratory distress syndrome, asthma, cardiovascular disease, rheumatological condition, chronic obstructive pulmonary disease, diabetes mellitus, hepatitis C, HIV, irritable bowel disease, neurological disorder, osteoporosis, peptic ulcer disease, prior malignancy, psychiatric condition, thyroid disease, tuberculosis, upper gastrointestinal disease, visual impairment, hearing impairment, disk degeneration and obesity), tumor primary, associated dates of diagnosis, first ICI, AE type and severity. Lung cancer was inclusive of both non-small-cell and small-cell carcinoma for this study.

### Definition of NAEs

Neurological adverse events (NAEs; primary outcome) were determined by retrospective review of patients’ problem lists, clinic notes, and hospitalization notes. For potential NAEs noted in problem lists, independent coders evaluated treatment and assessment notes to confirm the event as a NAE. Free text entries by nursing staff for the associated clinic visit or hospitalization were also evaluated using natural language processing to identify known phrasing associated with NAEs. If such language was identified, the event was categorized as a NAE. Specific terms (e.g., ICI encephalitis) were then grouped into the broader categories of central and peripheral NAEs following confirmation performed by the Vasta Global group. Peripheral NAE terms for primary complaints included neuropathic, eye, peripheral, foot drop, myasthenia gravis, ocular, optic and numbness. Central NAE terms included hypopituitarism, hypophysitis, headache, migraines, and dizziness. Fatigue was not included as a NAE, given the non-specific nature of the complaint and its higher prevalence of driving associations. Although headache and dizziness/syncope are also relatively nonspecific, they were considered localizable to the CNS and included, as previous studies have categorized [15].

### Definition of adverse event grade

Adverse event grading was defined using the Common Terminology Criteria for Adverse Events (CTCAE) supported by the National Cancer Institute (NCI) [14,16]. Grade was determined by data abstractors previously mentioned. Grade 1 was defined as asymptomatic or mild with little to no impairment; intervention was not indicated. Grade 2 was defined as moderate with limiting age-appropriate instrumental activities of daily living; intervention was indicated. Grade 3 was defined as severe and limiting activities of daily living or hospitalization was indicated. Grade 4 is defined as life-threatening and requiring urgent interventions. For this study, grades 1–2 were considered ‘low’ grades as they did not require hospitalization, while grades 3–4 were considered ‘high’ grades requiring a higher level of care.

### Statistics

SAS version 9.4 (NC, USA) was used for all analyses. Data were described as frequencies with percentages, medians with interquartile ranges, or means with standard deviations (SD) as appropriate depending on variable type and distribution. Unadjusted comparisons were made between continuous variables with a two-sample t-test or Wilcoxon rank sum, depending on the distribution. Categorical variables were compared using either chi-square or Fisher’s exact test. Logistic regression models were built to assess correlation with events. Covariables of interest (e.g., age, race/ethnicity, tumor primary, BMI, neurological comorbidities) were considered a priori, with these variables first evaluated for modeling significance defined as p ≤ 0.1. Selection models were then built to analyze potential risk factors (described in the *Study variables* section) and associated odds of a NAE. Models were run in a stepwise bidirectional method with inclusion set at p ≤ 0.4 and retention set at p ≤ 0.2. Kaplan-Meier curves were produced for all unadjusted survival data. Statistical significance was defined as p < 0.05.

## Results

### Population demographics

The final cohort included 3137 patients (Table 1 for characteristics). The cohort had an average age of 63 years old (SD = 12), and the majority identified as male (1894, 60%). Most patients (2653; 85%) identified as white or Caucasian. The primary cohort included many types of primary malignancies. lung cancer was the most common primary (1406; 45%), followed by melanoma (446; 14%). Other tumor primaries with independent incidence <5% in this cohort included breast, cervical, cutaneous squamous, endometrial, esophageal, gastric, hepatocellular, Hodgkin lymphoma, Merkel cell, urothelial.

**Table 1.** Patient characteristics for the entire cohort stratified by adverse event type.

	Total (N = 3,137)	CNS NAE (n = 23)	PNS NAE (n = 31)	Other irAE (n = 1,122)	None (n = 1,961)
**Age (years), mean (std)**	63.3 (12.0)	62.7 (9.1)	57.2 (14.0)	64.4 (11.8)	62.8 (12.2)
Missing	32	0	0	3	26
**Sex (female)**	1243 (40.03%)	7 (58.33%)	7 (41.18%)	297 (41.89%)	769 (39.74%)
**Race**					
White/Caucasian	2653 (85.44%)	11 (91.67%)	14 (82.35%)	630 (88.86%)	1618 (83.62%)
Black/African–American	361 (11.63%)	1 (8.33%)	2 (11.76%)	55 (7.76%)	260 (13.44%)
Other	91 (2.93%)	0 (0.00%)	1 (5.88%)	24 (3.39%)	57 (2.95%)
Missing	32	0	0	3	26
**BMI – categorical**					
Underweight	365 (11.76%)	1 (8.33%)	1 (5.88%)	47 (6.64%)	279 (14.43%)
Normal	1249 (40.25%)	3 (25.00%)	6 (35.29%)	261 (36.86%)	795 (41.11%)
Overweight	784 (25.27%)	3 (25.00%)	7 (41.18%)	200 (28.25%)	454 (23.47%)
Obese	705 (22.72%)	5 (41.67%)	3 (17.65%)	200 (28.25%)	406 (20.99%)
**Comorbidities**					
Neurologic	323 (10.30%)	5 (21.74%)	1 (3.23%)	100 (8.91%)	217 (11.07%)
Visual impairment	836 (26.65%)	6 (26.09%)	9 (29.03%)	316 (28.16%)	505 (25.75%)
Auditory impairment	365 (11.64%)	1 (4.35%)	3 (9.68%)	150 (13.37%)	211 (10.76%)

Categorical variables reported as count and frequency, missing values excluded from percentages.

Continuous variables reported as mean and standard deviation.

Race was self-identified and reported by the patient.

Neurological comorbidities include: Cerebrovascular accident, transient ischemic attack, hemiplegia, dementia, other.

CNS: Central nervous system; irAE: Immune-related adverse event; NAE: Neurological adverse event; PNS: Peripheral nervous system.

### Incidence & types of neurologic adverse events

Among these 3137 patients, 1176 (38%) reported at least one immune-related adverse event. Of a total of 1176 who reported any immune-related adverse event, a small number (54, 3%) were classified as NAEs. No patients reported more than one NAE.

Among NAEs, the majority involved the peripheral nervous system (31, 57%) as opposed to the central nervous system (23, 43%). Among central NAEs, there were similar proportions of low-grade (12, 52%) and high-grade severities (11, 48%). Peripheral NAEs also had similar proportions of low-grade (17, 55%) and high-grade severities (14, 45%). Other adverse events occurred in 1121 (36%) of all patients, of which the majority were low-grade (711, 63%) as opposed to high-grade severity (410, 37%).

### Correlates of neurologic adverse events

Six (11%) of patients experiencing a NAE had pre-existing neurological comorbidities (inclusive of cerebrovascular accident, transient ischemic attack, hemiplegia, dementia and or others unspecified). Most high-grade central NAEs occurred in patients with melanoma or renal primaries (four cases of melanoma and four renal primary tumors). In contrast, most high-grade peripheral NAEs occurred in patients with lung or melanoma primaries (six lung, four melanoma). Tumor primary was significantly associated with all distributions of NAEs, with a predominance of tumor primaries being melanoma or renal (Table 3).

**Table 2.** Adverse events and sub-types by tumor primary.

	Total (N = 3137)	CNS NAE^†^^,^^‡^ (n = 23)	PNS NAE^†^ (n = 31)	Other irAE (n = 1122)	None (n = 1961)
**Tumor primary**					
Lung	1406 (44.82%)	3 (13.04%)	11 (35.48%)	440 (39.22%)	952 (48.52%)
Melanoma	446 (14.22%)	9 (39.13%)	8 (25.81%)	256 (22.82%)	173 (8.82%)
Renal	196 (6.25%)	6 (26.09%)	5 (16.13%)	90 (8.02%)	95 (4.84%)
Other	1089 (34.71%)	5 (21.74%)	7 (22.58%)	336 (29.95%)	742 (37.79%)

† Significant (p < 0.05) compared with “None”.

‡ Significant (p < 0.05) compared with “Other”.

All tests performed using Fisher’s exact test.

‘Other Tumor primary’ includes: breast, cervical, cutaneous squamous, endometrial, esophageal, gastric, hepatocellular, Hodgkin lymphoma, Merkel cell, urothelial.

CNS: Central nervous system; irAE: Immune-related adverse event; NAE: Neurological adverse event; PNS: Peripheral nervous system.

**Table 3.** Exploratory modeling, stratified by event type.

CNS NAE		PNS NAE	
Variable	OR (95% CI)	Variable	OR (95% CI)
**Tumor primary (reference Other)**		**Tumor primary (reference Other)**	
Lung	0.49 (0.12–2.05)	Lung	1.17 (0.45–3.03)
Melanoma	3.95 (1.31–11.95)	Melanoma	3.44 (1.22–9.71)
Renal Cell Carcinoma	6.44 (1.92–21.58)	Renal Cell Carcinoma	4.66 (1.45–15.02)
**Comorbidities**		**Comorbidities**	
Neurological	2.96 (1.05–8.36)	Neurological	4.77 (0.59–38.28)
Obesity	2.11 (0.90–4.95)	Irritable Bowel Disease	0.26 (0.04–1.94)
Cardiovascular disease	0.53 (0.20–1.39)	Obesity	0.20 (0.05–0.86)
		Psychiatric	1.80 (0.87–3.71)
		Peptic Ulcer Disease	3.76 (1.10–12.88)

Neurological comorbidities include cerebrovascular accident – transient ischemic attack – hemiplegia – dementia – and others.

Cardiovascular comorbidities include coronary vascular disease – prior myocardial infarction – peripheral arterial disease.

Peptic ulcer disease comorbidity includes any history of treatment.

Psychiatric comorbidity includes requiring psychiatric consult – medication – or other treatment.

‘Other tumor primary’ includes: breast, cervical, cutaneous squamous, endometrial, esophageal, gastric, hepatocellular, Hodgkin lymphoma, Merkel cell, urothelial.

CNS: Central nervous system; irAE: Immune-related adverse event; NAE: Neurological adverse event; PNS: Peripheral nervous system.

### Correlates of neurological adverse events

Table 1 presents hypothesized risk factors considered a priori for predicting either central or peripheral NAEs, except for tumor primary, presented separately in Table 2. Final prediction models based on exploratory stepwise analyses are shown in Table 3. In the final model for all grade central NAEs, the only a priori variables retained were tumor primary and neurological comorbidity; age, sex and race did not meet model inclusion criteria. Neurological comorbidities were significantly associated with central NAE (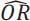 = 2.96; 95% CI: 1.05–8.36). Despite a similarly indicated association neurological comorbidities were not significantly associated with peripheral NAE as described in Table 3. When compared with all other tumor primaries, renal cell carcinoma (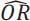 = 6.44; 95% CI: 1.92–21.58) and melanoma (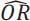 = 3.95; 95% CI: 1.31–11.95) were associated with increased odds of a central NAE. Neurological comorbidity was also associated with increased odds of a central NAE (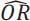 = 2.96; 95% CI: 1.05–8.36). Other exploratory variables of interest that met model inclusion included obesity and cardiovascular disease, but these variables were not significantly associated with central NAE.

Regarding exploratory modeling of all grade peripheral NAEs (Table 3), tumor primary and neurological comorbidities remained the only a priori variables of interest to meet model inclusion. Renal cell carcinoma (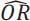 = 4.66; 95% CI: 1.45–15.02) and melanoma (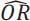 = 3.44; 95% CI: 1.22–9.71) were associated with increased odds of a peripheral NAE compared with ‘other’ tumor primaries. Though obesity, neurological comorbidities, irritable bowel disease (IBD), and psychiatric comorbidities also met model inclusion criteria, these factors were not significantly associated with peripheral NAEs. Peptic ulcer disease (PUD) was also significantly associated with peripheral NAEs (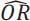 = 3.76; 95% CI: 1.10–12.88)

### Time to event results

**Figure 1.**
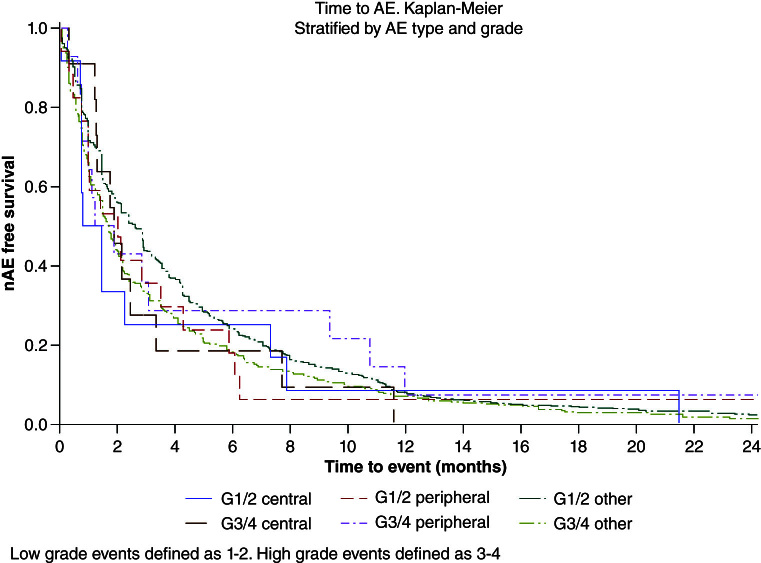
Kaplan-Meier plot for time to each neurological event by severity and type.

Time to event for irAEs and central and peripheral NAEs is presented graphically via Kaplan-Meier curves in Figure 1. Among patients who experienced ICI-related adverse events, the time from the first dose of ICI to experiencing an adverse event was 2.1 months (IQR: 0.8–5.1). The median time to any NAE was 1.6 months (IQR: 0.7–4.2); the time to a grade 1–2 event was 1.1 months (IQR: 0.7–4.7) for CNS and 2 months (IQR: 0.9–4.2) for PNS. Time to a grade 3–4 event was 1.8 months (IQR: 1.2–3.3) for CNS and 1.5 months (IQR: 0.7–4.2) for PNS. Time-to-event data is further described in Table 4 to present the stratification by adverse event type and grade.

**Table 4.** Survival data stratified by event type and grade.

	Grade 1 or 2			Grade 3 or 4			
	CNS NAE (n = 12)	PNS NAE (n = 17)	Other irAE (n = 712)	CNS NAE (n = 11)	PNS NAE (n = 14)	Other irAE (n = 410)	None (n = 1961)
**Time ICI to irAE (months)**	1.1 (0.7, 4.7)	2 (0.9, 4.2)	2.5 (0.9, 5.7)	1.8 (1.2, 3.3)	1.5 (0.7, 9.3)	1.6 (0.7, 4.2)	NA

Continuous variables reported as median [IQR].

CNS: Central nervous system; irAE: Immune-related adverse event; ICI: Immune checkpoint inhibitor; NAE: Neurological adverse event; PNS: Peripheral nervous system.

## Discussion

In our large retrospective cohort, we found an incidence rate of 1.72% for neurologic adverse events. There were slightly more NAEs with PNS involvement than CNS involvement (57 vs 42%). Most NAEs occurred in patients with melanoma, lung, and renal primaries. Potential associations with central NAEs included tumor primary (specifically renal and melanoma) and neurological comorbidities. Potential associations with peripheral NAE included tumor primary (melanoma, renal) and peptic ulcer disease (PUD). Time to event was broadly similar for central and peripheral NAEs and was also similar to timing for other AEs.

Within our large cohort, the incidence of NAEs remained consistent with previous estimations of 1.0–2.0% at 1.72% for all grade events but much lower than other studies estimating rates of 7 or 12%. The prevalence of high-grade NAEs in this study was 25 (0.8%), in alignment with previous research estimating that these events occur in less than 1% of patients receiving ICI [12,13]. However, these may be underestimates given that NAEs have been challenging to characterize and fully describe. Further, it remains possible that this variance in incidence estimation remains an element of reporting bias as many of the higher prevalence estimates have occurred in the FDA reporting database specific to events or drug trials with closer monitoring than in a more traditional clinical setting.

We found that peripheral NAEs were more common than central NAEs, experienced by 0.98 versus 0.73% of patients, respectively. However, they remained relatively much closer than previously shown in previous cohorts by Larkin *et al.* and Möhn *et al.* [7,15]. This may be due to the generally inclusive definition of central events for this study, including headaches and dizziness, though equally generalized terms of numbness were included by natural language processing of chief complaints related to the peripheral nervous system. Although estimates must be interpreted cautiously given very small cell sizes, we found a relatively equal distribution of low- and high-grade events for central and peripheral NAEs. Low-grade events remained more common, but only slightly, with 1 and 3 more events in central and peripheral low-grade events, respectively. This is opposed to the 40% increased incidence of low-grade events among other types of adverse events.

In exploratory modeling, our study identified several potential risk factors for NAEs requiring further analysis. For central NAEs, the only variables considered a priori that met model inclusion were tumor primary and neurological comorbidities. Tumor primary (specifically renal cell carcinoma and melanoma) was most strongly associated with central NAEs. Among peripheral NAEs, again, the only variables of interest a priori to meet model inclusion were tumor primary and neurological comorbidity. Again, renal cell carcinoma and melanoma were among the strongest associations. Neurological comorbidities being significantly associated with central NAE may be suggestive of a predisposition due to pre-existing cellular damage. Though it was positively associated with peripheral NAE but not significant is likely due to heterogeneity of comorbidities as represented in the wide confidence interval for peripheral events (95% CI: 0.59–38.28). Interestingly, peptic ulcer disease (PUD), a variable included on an exploratory basis, was associated with increased odds of peripheral NAEs. Although these analyses must be interpreted cautiously, especially given their experimental nature and small cell sizes (reflected in wide confidence intervals), these results nevertheless point to potential risk factors requiring further study. Other possible risk factors identified in exploratory analyses included additional comorbidities (IBD, obesity and psychiatric disorders).

Questions remain, though, as to the underlying mechanism behind NAEs. We found that NAEs were mostly present among patients with specific tumor primaries (e.g., melanoma, renal), consistent with previous research [10]. It is possible that NAEs were mostly among patients with these primaries because they were the most common primaries in our database. Still, these primaries are also classically favored to metastasize to the brain. Although we could not directly test theories regarding mechanisms underlying NAEs in the present study, these results may shed light on the underlying etiology. There are two prevailing theories to explain how some individuals experience NAEs, the first being hidden autoimmunity [17]. This theory suggests patients have an existing subclinical autoimmune disease being masked before ICI treatment. The second mechanism theory for NAE is molecular mimicry: the antigen of tumor cells has functionally identical epitopes to the subsequently targeted neurons [18]. Given the risk factors identified, these results indirectly support molecular mimicry and hidden autoimmunity.

Regarding tumor primary, the fact that three highly prevalent tumor primaries (renal, melanoma, lung) were associated with both central and peripheral NAEs supports a potentially similar epitope between tumor and normal tissue. Though lung cancer was one of the most prevalent primaries, the relative incidence among NAEs was lower than the comparative incidence among no irAE, as opposed to melanoma and renal cell carcinoma, which were significantly associated with central and peripheral NAEs.

Exploratory analyses of comorbidities may support theories of a hidden autoimmunity etiology. With neurological comorbidities meeting inclusion criteria in both models and significance among central NAEs, this also has the potential to support molecular mimicry. With pathogenesis such as a CVA causing destruction and shedding of potential antigens or peptic ulcer disease causing a breakdown in the mucosal barrier to the blood, there could be a subclinical autoimmunity that is brought to the forefront by the ICI. Finally, it is interesting to consider the seemingly protective effect of obesity regarding peripheral NAEs. The obesity paradox has been noted numerous times in previous literature that has not yet come to a conclusion [19,20]. It is entirely possible this remains a confounder either by unmeasured confounding or other possibilities such as NAE neuropathies being underreported since being masked by existing neuropathies. An additional consideration for the future may include previous or neoadjuvant cytotoxic chemotherapy given potential cell lysis and epitope release. Further studies with larger samples and prospective data are needed to understand these potential risk factors and mechanisms further.

We found that time to event was variable for NAEs, but medians were similar in timing for other AEs (1.6 months among NAEs compared with 2.1 months for other adverse events). Additionally, there was no clinically meaningful difference between the adverse event subtype and event grade. This supports a window of increased surveillance and follow-up within the first 1 to 2 months of initiating ICI to monitor adverse events. As further supported in Figure 1, an initial sharp incidence exists after about five months. While this may be impacted by survivorship bias, it suggests an unmasking or initially pre-disposed mechanism for the adverse events.

As the underlying mechanism of NAE is further explored and understood, understanding clinical impact and monitoring for NAEs continue to be necessary. NAEs remain difficult to diagnose as they are diagnoses of exclusion. To our knowledge, the present study is the first to stratify results by central and peripheral NAEs, extending previous literature, such as a recent study using the FAERS database, which grouped NAE etiology (e.g., Guillain-Barre syndrome) rather than peripheral or central events [10]. In contrast to this study by Mikami and colleagues, we found no association between demographics and NAEs, although we replicated their findings regarding an association between melanoma and NAEs. Further, they found an association between dual ICI therapy and fatal NAEs. Though we could not explore dual ICI therapy as a risk factor for NAE in our study, this is a standard regimen in the treatment naive, metastatic renal cell carcinoma [21]. With this combination of evidence, further study into mechanisms of NAEs will be best served, focusing on melanoma and renal primaries. While lung cancer accounted for a meaningful number of events, this was likely driven by its higher prevalence and may be more challenging to study rare events.

The results of this study should be considered within the context of its limitations. First, it is a retrospective study and is thus subjected to reporting bias. Patients who experience adverse events may be more likely to have complete data reporting by increased medical contact. Systematic chart review by VASTA was intended to combat this as they generated the data systematically for the database rather than for the intent of this work on adverse events. Though adverse events were stratified by peripheral and central nervous system, this study did not analyze events by their specific etiology (such as neuropathy, myopathy, etc), potentially overlooking more granular associations. As stated previously, model building for potential risk factors was exploratory. Though there was a staged approach to prioritize a-priori associations, the subsequent exploratory modeling may be subjected to type 1 error. The specific ICI that each patient received or in combination was not available; thus, tumor primary may be influenced by unmeasured confounding in the context of standard ICI received for each. Finally, given the continued uncertainty of these events’ mechanisms, there is the possibility of unmeasured confounding. Nevertheless, given the strengths of this study (larger sample size, real-world clinical data), results point to key areas requiring further study and thus shed light on the nature and timing of NAE among patients treated with ICI.

## Conclusion

Neurological adverse events remain a meaningful and important condition for physicians to vigilantly monitor patients as the indications for ICI expand [22].

Immunotherapy is an impressive and pivotal emergence of cancer treatment, and as it expands, so will the rates of adverse events. Neurological adverse events remain rare but disproportionally severe. Associations with tumor primary suggest molecular mimicry may play a pivotal role in understanding the mechanism of these events. Further study in the peak window of these events, approximately 2 months after starting treatment, may shed light on the driving etiologies and their prevention.

## Key points

- Neurological adverse events incidence ranged from 1–2% among a large cohort.
- Time-to-event data was consistent with all other ICI-related adverse events.
- Consistent with previous research, NAEs were most commonly seen in melanoma and renal cancers.

**Summary points**

- Neurological adverse events have historically been infrequent and challenging to comprehensively characterize in prior research.
- Previous estimates of neurological adverse event incidence have shown considerable variability, ranging from less than 1% in case series to approximately 7% in larger cohorts.
- In our study involving 3137 patients, we identified a neurological adverse event incidence of 1.7%, falling within the spectrum of prior estimates.
- The median time from the first immune checkpoint inhibitor (ICI) dose to a neurological adverse event was approximately 6 to 8 weeks, aligning with the timing observed for other adverse events.
- Baseline neurological comorbidities, encompassing conditions like CVA, TIA, hemiplegia and dementia, exhibited discordance between neurological adverse event types and other events, emerging as significant predictors in our modeling.
- Tumor primary was notably associated with the occurrence of neurological adverse events, with melanoma and renal cell carcinoma being the most frequently linked tumor types.
- Melanoma demonstrated an adjusted hazard ratio (95% CI) of 3.95 (1.31–11.95) for central neurological adverse events and 3.44 (1.22–9.71) for peripheral events.
- Renal cell carcinoma exhibited an adjusted hazard ratio (95% CI) of 6.44 (1.92–21.58) for central adverse events and 4.66 (1.45–15.02) for peripheral adverse events.
- Given that a majority of neurological adverse events occurred within the initial two months and considering the identified risk factors, further investigation is warranted to gain a more nuanced understanding of these events.

## Data sharing statement

Data will be made available per written request to the corresponding author. The data used was pre-existing prior to the study and not generated from a clinical trial.
